# Tissue Regeneration with Gelatine/Polysaccharide Derived Hydrogel Scaffolds: From Formulation to In Vivo Efficacy

**DOI:** 10.3390/gels9090744

**Published:** 2023-09-13

**Authors:** Jing Li, Keying He, Qian Xu

**Affiliations:** 1 Department of Stomatology, Huadong Hospital, Fudan University, Shanghai 200437, China; lijing_@fudan.edu.cn; 2 Woundhealing (Hangzhou) Biotechnology Co., Ltd., Hangzhou 310018, China; keyinghwh@163.com; 3 School of Pharmaceutical Sciences, Wenzhou Medical University, Wenzhou 325015, China

**Keywords:** gelatine, polysaccharide, hydrogel formulation, vascularisation, tissue regeneration

## Abstract

Combinations of different biomaterials with certain formulations may lead to improved properties and have significant potential for use in tissue regeneration applications. However, previously reported studies comparing biomaterials often suffered from inconsistent processing methods or inadequate comprehensive application research, hindering a comprehension of their efficacy in tissue engineering. This report explores the significance of screening the combination of gelatine with polysaccharide materials, specifically hyaluronic acid (HA) and carboxymethyl cellulose (CMC), using the same crosslinking method used for tissue regeneration. Hydrogel scaffolds (Gel/HA and Gel/CMC) at various concentrations were developed and characterized to assess their physiochemical properties. The results demonstrated that the hydrogels exhibited desirable mechanical properties, appropriate swelling behaviour, suitable porosity, and excellent cytocompatibility. In particular, the Gel1HA1 and Gel1CMC1 hydrogels showed remarkable cellular proliferation and aggregation. Further, we performed animal studies and explored the tissue regeneration effects of the Gel1HA1 and Gel1CMC1 hydrogels. Both hydrogels exhibited an accelerated wound closure rate and promoted vessel formation in a rodent full-thickness skin excisional model. Additionally, the subcutaneous implantation model demonstrated the induction of angiogenesis and collagen deposition within the implanted hydrogel samples. Overall, the hydrogels developed in this study demonstrated promising potential for use in the regeneration of soft tissue defects and this study emphasizes the significance of screening biomaterial combinations and formulations for tissue regeneration applications.

## 1. Introduction

Tissue engineering is a promising field that aims to develop functional tissues for the regeneration and repair of damaged or diseased tissue [1]. Biomaterials, with their unique properties, have emerged as valuable tools in this pursuit. Among many options, hydrogel scaffold has been recognized as a promising option due to the ease of hydrogel formation and the material’s unique properties [2]. Hydrogels are polymer networks that are predominantly composed of water (approximately 90% of its weight). Hydrogels are typically soft and flexible, affording it to be well-suited for the treatment of soft tissues, like skin, oral mucosa [3]. These viscoelastic materials have shown themselves to be effective in tissue regeneration applications in recent years. 

Numerous biomaterials have been manufactured for hydrogel fabrication in attempts to regenerate different tissues and organs, including naturally derived materials (hyaluronic acid, collagen, gelatine, etc.), synthetic materials (poly(ethylene glycol), poly-l-lactic acid, etc.), and hybrid hydrogels that combine both synthetic and natural materials [1,4,5]. Hydrogel formation can be achieved through either physical (noncovalent assembly) or chemical gelation (covalent crosslinking) [6]. Physically crosslinked hydrogels exhibit the advantages of easy processing, no requirement for crosslinkers, and being easy to reverse. However, physical hydrogels are heterogeneous and have poor mechanical properties, which may limit their tissue engineering applications. Chemically crosslinked hydrogels can be obtained via covalent bond formation through chemical reactions, radical polymerisation, photo-crosslinking, etc. Such hydrogels have higher mechanical strength and homogenous structure. However, there might be residual chemical crosslinkers, organic solvents, or photo-initiators that may cause poor biocompatibility. Thus, it is important to investigate a biocompatible and chemical crosslinking approach for hydrogel creation. There are a number of key properties that are essential when designing hydrogels for tissue engineering applications, such as biocompatibility, biodegradability, mechanical strength, and porosity [7]. It is also important to understand and modulate these characteristics as they influence the utility of hydrogels for biological and biomedical applications. The combination of these two materials offers a compelling avenue for enhancing tissue regeneration [8]. However, the successful development of biomaterial-based therapies requires a comprehensive screening process to identify optimal combinations and formulation. 

To create effective hydrogel scaffolds, it is crucial to carefully select biomaterial component based on their fundamental bioactivity and characteristics. Among the various biomaterials, natural biomaterials, such as collagen-derived materials and polysaccharides, have garnered significant attention due to their biocompatibility, biodegradability, and ability to mimic the natural extracellular matrix (ECM) [9,10]. Gelatine, a collagen-derived material, possesses excellent biocompatibility compared with collagen and exhibits a desirable ability to form hydrogel scaffolds [10,11,12]. However, hydrogels formed by gelatine alone have limitations in terms of mechanical strength, degradability, porosity, and bioactivity, necessitating the exploration of combinations with other materials. Hyaluronic acid (HA) is a widely studied biomaterial in tissue engineering [13,14]. It is a natural polysaccharide found in the extracellular matrix and has exceptional biocompatibility and bioactivity. HA can retain water, promoting cell adhesion, migration, and proliferation. Combining gelatine with HA has been investigated in previous studies to enhance mechanical properties, improve cell behaviour, and promote tissue regeneration [15,16,17]. Carboxymethyl cellulose (CMC), a derivative of cellulose, also possesses carboxyl groups that can crosslink with amine groups in gelatine [18]. CMC has excellent water retention capacity, biodegradability, and film-forming properties. By incorporating CMC into gelatine-based scaffolds, researchers have achieved improved mechanical strength, controlled degradation rates, and enhanced cell adhesion and proliferation [19]. 

Researchers have studied the physiochemical properties of materials comprising gelatine and polysaccharides using different crosslinking approaches and explored their potential in tissue engineering [19,20,21,22,23]. Such combinations have demonstrated improved mechanical properties, tuneable degradation rates, increased cell adhesion, and enhanced tissue regeneration potential. These findings also highlighted the importance of screening appropriate biomaterial components and formulations to achieve optimal scaffold performance for successful tissue regeneration. However, prior studies often suffered from inconsistent processing methods or a lack of comprehensive application research, hindering a comprehensive understanding of their efficacy in tissue engineering [20,24]. Therefore, it is necessary to compare and screen biomaterial combinations and formulations on the basis of the same crosslinking method and explore their potential applications through systematic biological analysis [2,25].

In this report, we investigated the significance of screening the combination of gelatine and polysaccharide materials, specifically HA and CMC, for their use in tissue regeneration. To achieve this objective, we developed Gel/HA and Gel/CMC hydrogel scaffolds using varying concentrations. We employed 1-ethyl-3-[3-dimethylaminopropyl] carbodiimide hydrochloride (EDC) to effectively crosslink gelatine with either HA, CMC, or gelatine alone. The resulting hydrogels displayed proper mechanical properties, swelling behaviour, and porosity, demonstrating excellent cytocompatibility. Furthermore, the Gel1HA1 and Gel1CMC1 hydrogels exhibited remarkable cellular proliferation and aggregation. To explore the tissue regeneration effect of the hydrogels, we first used a full-thickness skin excisional wound model in vivo as a representative tissue defect model to test tissue repair efficacy. Furthermore, we performed a subcutaneous implantation model in vivo to explore the tissue integration and regeneration effect of the hydrogels. 

## 2. Results and Discussion

### 2.1. Fabrication of Gelatine-Based Hydrogel Scaffolds and Characterisation

**Figure 1:**
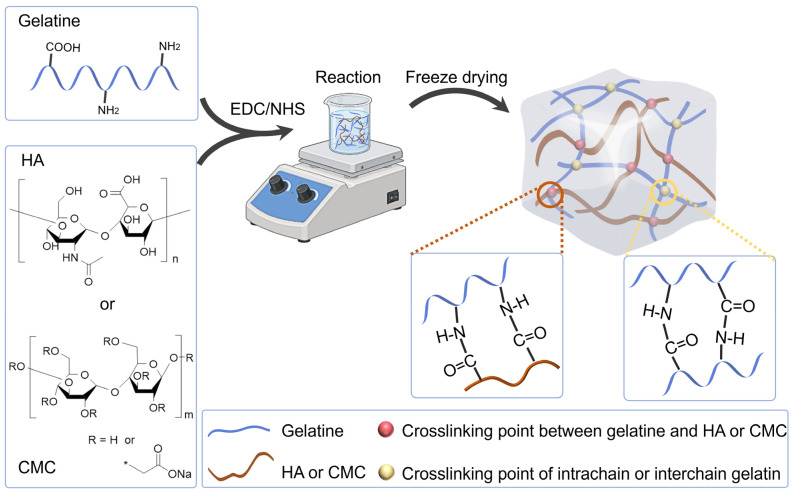
Schematic of the synthesis of hydrogel scaffolds. Gelatin and HA or CMC were mixed and reacted by EDC/NHS-mediated crosslinking in a beaker while stirring. Subsequently, the reaction solution underwent freeze drying to produce the scaffold.

Various gelatine-based hydrogels have been extensively developed and studied for use in tissue engineering due to their feasible fabrication methods and advantageous formulation. In general, it is considered appropriate to utilize a relatively low concentration of gelatine in order to fabricate soft tissue engineering scaffolds. By incorporating an additional material that can crosslink with the amine or carboxyl groups present in gelatine, the mechanical properties, degradability, and porosity of the resultant hydrogels will be adjusted, effectively overcoming the limitations associated with hydrogel scaffolds composed solely of gelatine. We selected hyaluronic acid (HA) and (CMC) as representative polysaccharides, each possessing carboxyl groups, for effective crosslinking with the amine groups present in gelatine using the EDC method (Figure 1). As a gelatine molecule has multiple carboxyl groups and amine groups, both intermolecular cross-linking and intramolecular cross-linking of gelatine could also occur, as shown in Figure 1. 

**Figure 2:**
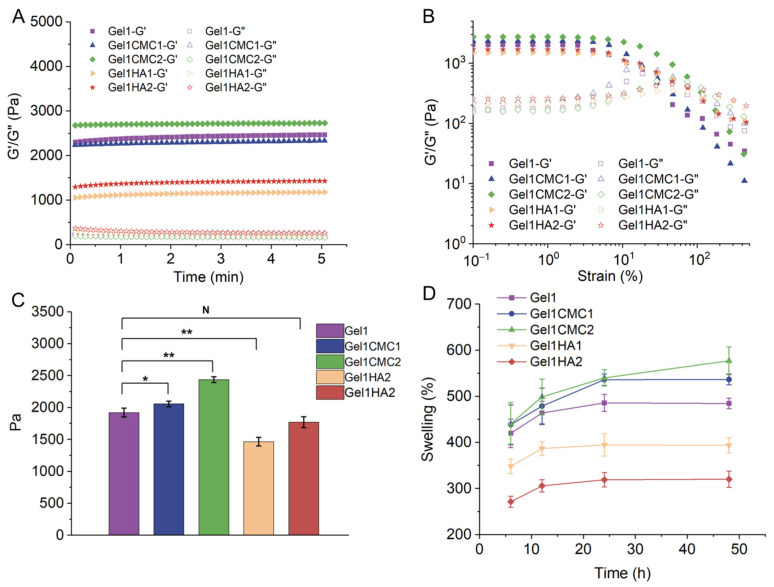
Characterisation of the hydrogels. (**A**). Time sweep assessment of the hydrogels. (**B**). Strain sweep assessment of the hydrogels. (**C**). The storage modulus of the hydrogels. (**D**). Swelling behaviour of the hydrogels. * *p* < 0.05; ** *p* < 0.01; ^N^ *p* > 0.05.

The mechanical properties of the hydrogels were evaluated and presented in Figure 2. A fixed concentration of gelatine at 1.58% (*w*/*v*) was used for all the hydrogels. CMC or HA was chemically incorporated at 0.1% and 0.2%, respectively, and crosslinked with 1.58% gelatine. As the concentration of CMC or HA increased, the storage modulus of the resulting hydrogels also increased, as shown in Figure 2A,C. There is no significant difference between the various hydrogels. Additionally, a strain sweep test was conducted over the strain range between 0.1% and 100% (Figure 2B). The mechanical properties of a scaffold are essential characteristics for a hydrogel designed for tissue engineering. A hydrogel should have excellent mechanical strength in order to temporarily replace those characteristics the tissue is lacking and protect the surrounding tissue without adding extra pressure. It should also have sufficient strength and mechanical stability to withstand its implantation and the typical loading conditions of in vivo tissue stresses. Moreover, when used in cell delivery, a hydrogel should have appropriate mechanical properties to regulate the cell behaviour for the proposed therapeutic targets [26]. The hydrogels developed in this study are within the range promoting soft tissue mechanical properties, indicating that they will not influence the surrounding cellular behaviour of normal tissue after application.

The swelling behaviour of the hydrogels over 48 h is shown in Figure 2D. All hydrogels exhibited a rapid increase in swelling degree within the first 24 h. Specifically, Gel1 and Gel1CMC2 hydrogels showed continuous swelling throughout the 48 h period, while the remaining three hydrogels reached swelling equilibrium within 24 h. The enhanced swelling performance of CMC in Gel1CMC2 could account for this observation. The swellability of a hydrogel is a crucial parameter when considering the design of a hydrogel matrix for tissue engineering. An ideal matrix should possess the capacity to absorb wound fluids, facilitating nutrient and waste diffusion. However, excessive swelling can lead to wound maceration and impose compressive stress on the surrounding tissue, potentially causing further damage [27]. Therefore, careful screening of the composite hydrogel formulation is essential before considering its application in real clinical settings. 

**Figure 3:**
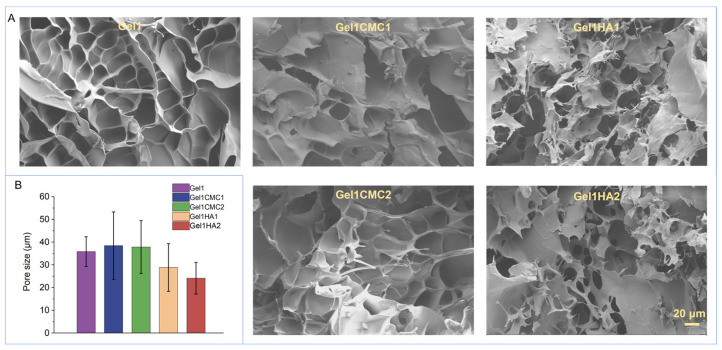
Morphology of the hydrogel scaffold. (**A**). SEM images of the hydrogels. Scale bar: 20 μm. (**B**). Average pore size.

Morphological analysis was performed using SEM detection, as depicted in Figure 3A. All hydrogels exhibited an evident pore structure. The average pore sizes were measured as follows: 35.8 ± 6.6 μm for Gel1, 38.5 ± 14.8 μm for Gel1CMC1, 37.9 ± 11.6 μm for Gel1CMC2, 28.9 ± 10.5 μm for Gel1HA1, and 24.1 ± 7.0 μm for Gel1HA2. Gel1 hydrogel showed the smoothest surface morphology. Increased HA concentration slightly decreased the pore size. The porosity and pore size of a scaffold are also important factors to consider in tissue engineering. These parameters significantly influence cell infiltration, nutrient transport, and waste removal, thereby impacting the overall performance of the scaffold in promoting tissue regeneration. The observed differences in pore sizes among the hydrogels underscore the successful outcome of screening the scaffold’s formulation to optimise its parameters for specific tissue engineering applications. 

### 2.2. In Vitro Cytocompatibility Assessment

**Figure 4:**
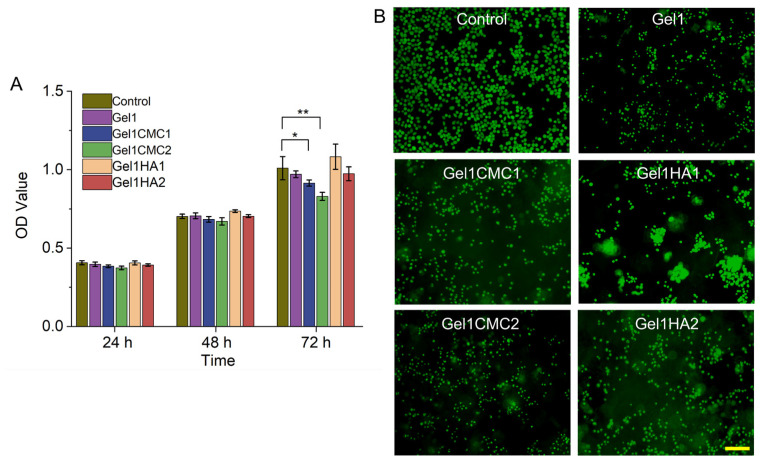
In vitro cell experiment. (**A**). CCK-8 test of the hydrogels. * *p* < 0.05, ** *p* < 0.01. (**B**). Representative fluorescent images of live/dead staining assay. Scale bar. 20 μm.

The cytotoxicity and proliferation effect of the hydrogels were assessed using a CCK-8 assay with 3T3 fibroblasts. The cells were seeded ono the surface of the gels to mimic the in vivo microenvironment of an implanted gel and replicate the natural progression of cell interactions that occur during the tissue regeneration process. Cells cultured with a full cell culture media were referred to as the “control” group. As shown in Figure 4A, the proliferative capacity of the cells increased in all hydrogel groups over 48 h. However, Gel1CMC1 and Gel1CMC2 exhibited a lower proliferation ratio at 72 h compared to the control group (full cell culture media). This effect could be attributed to the high osmotic pressure resulting from the high swelling ratio of both hydrogels. Conversely, the HA introduced into the hydrogels displayed a high proliferation rate. Furthermore, live/dead staining of the cells seeded in the hydrogels demonstrated that most of the cells remained viable within the hydrogel (Figure 4B). Interestingly, cells seeded in Gel1HA1 hydrogel displayed significant cell aggregation compared to the other hydrogel groups. This observation indicates that the formulation of Gel1HA1 facilitates cell proliferation and migration inside the hydrogel. As previously reported, the cells cultured as 3D aggregates can enhance the endogenous secretion of bioactive factors crucial for cell survival and cellular function, which ultimately improves therapeutic outcomes [28]. The phenomenon observed from our in vitro experiment highlights hydrogel’s potential as a viable candidate for addressing a cell delivery niche. 

These physiochemical and in vitro findings emphasise the distinctive attributes of Gel1HA1 and Gel1CMC1 hydrogels, suggesting their versatility for use in a range of biomedical applications beyond tissue regeneration. 

### 2.3. In Vivo Skin Wound Healing

**Figure 5:**
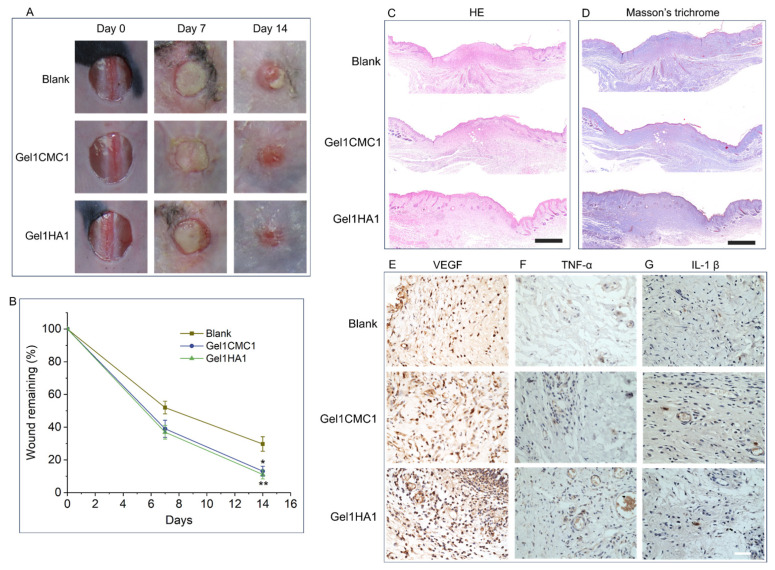
In vivo skin wound healing. (**A**). Gross images of wounds with different treatment groups. (**B**). Percentage of remaining wound area at different time points. * *p* < 0.05, ** *p* < 0.01. (**C**). HE staining of wound tissue on day 14. Scale bar: 1 mm. Masson’s trichrome staining of wound tissue on day 14. Scale bar: 1 mm. (**E**–**G**). Immunohistochemistry analysis of VEGF, TNF-α, and IL-1β. Scale bar: 20 μm.

To confirm the potential of the hydrogels prepared in this work, we first verified it using an excisional full-thickness skin wound model. Based on the physiochemical and in vitro results, Gel1CMC1 and Gel1HA1 were selected as the experimental group. As shown in Figure 5A, after 7 days of treatment, all wounds exhibited reduced size compared to the original wound areas. Subsequently, after 14 days of treatment, the wounds treated using Gel1HA1 (11.1 ± 2.7%) and Gel1CMC1 (13.1 ± 3.1%) demonstrated significantly faster healing compared to the untreated wounds (Blank) (29.7 ± 4.4%). No significance was observed between Gel1HA1 and Gel1CMC1 in the wound closure rate. To further evaluate wound regeneration efficacy, histological and immunochemical analyses were conducted. The HE staining assay revealed the formation of the regenerated epidermis and skin attachment. Collagen deposition in the healed wound was detected via Masson’s trichrome staining. As shown in Figure 5C,D, the untreated wounds showed a high amount of inflammatory cell infusion in the wound area and incomplete re-epithelisation. In contrast, both Gel1HA1- and Gel1CMC1-treated wounds showed complete re-epithelisation and new granulation formation. Intriguingly, the Gel1HA1-treated wound showed regenerated skin appendages, including hair follicles, and regular collagen deposition, indicating more comprehensive skin regeneration after the treatment of Gel1HA1. The capability of promoting angiogenesis was confirmed through VEGF staining of the tissue sections. The inflammation response was identified by the expression of inflammatory cytokines (TNF-α and IL-1β) in the positively stained cells. As shown in Figure 5E, the increased VEGF expression was observed in both Gel1CMC1- and Gel1HA1-treated wounds. The expression of TNF-α and IL-1β in all wounds were not significantly elevated (Figure 5F,G). These data demonstrate the remarkable wound-healing potential of Gel1HA1 and Gel1CMC1 hydrogels, suggesting their applicability for promoting skin tissue repair. 

**Figure 6:**
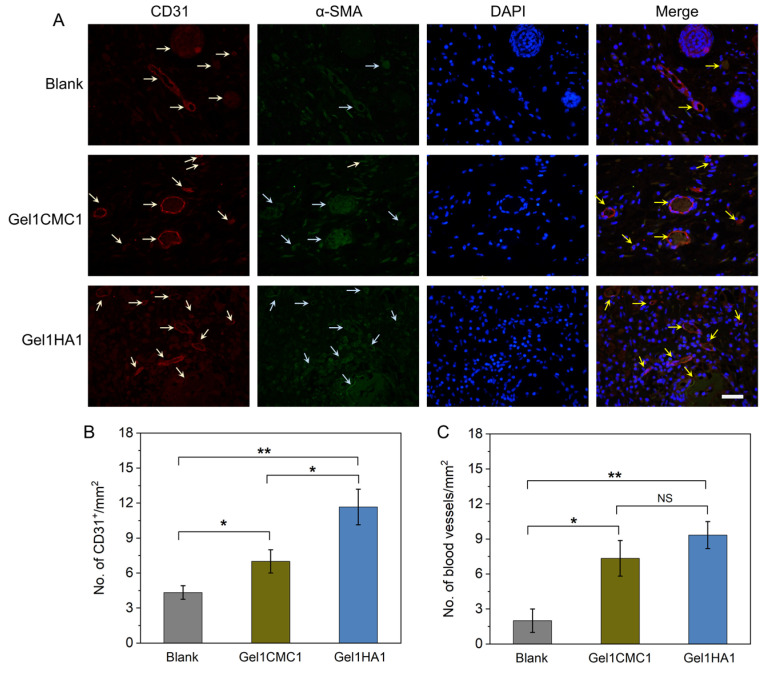
Vascularisation analysis of wound tissues. (**A**). Immunofluorescence of CD31, and α-SMA. Scale bar: 20 μm. (**B**). Quantitative analysis of CD31-positive stained immature vessels. (**C**). Quantitative analysis of both CD31- and α-SMA-positive stained mature vessels. * *p* < 0.05, ** *p* < 0.01, NS, no significant difference.

Angiogenesis plays critical roles in the wound healing process by facilitating the delivery of nutrients and oxygen to the injured tissue and draining out the wastes. We conducted an immunofluorescent assay to observe the CD31- and α-SMA-positive stained cells (Figure 6A). Furthermore, we quantified the CD31-positive and α-SMA positively staining cells to determine the presence of immature and mature vessels after treatment. The quantitative data showed that a significantly higher number of CD31 positively stained immature vessels were found in wounds treated using Gel1HA1 and Gel1CMC1 (Figure 6B). Additionally, a greater number of mature vessels were detected in wounds treated with Gel1HA1 and Gel1CMC1 compared to untreated wounds, as evidenced by the co-staining of both CD31 and α-SMA. No significant differences were found between Gel1HA1- and Gel1CMC1-treated wounds (Figure 6C). These data collectively indicate that the formulated hydrogels of Gel1HA1 and Gel1CMC1 accelerated skin wound closure rate, promoted angiogenesis, granular formation, and re-epithelialisation, and induced a neglectable inflammation response. Thus, such hydrogel formulations represent a promising new strategy for the treatment of skin wounds. 

### 2.4. In Vivo Hydrogel Intergration

**Figure 7:**
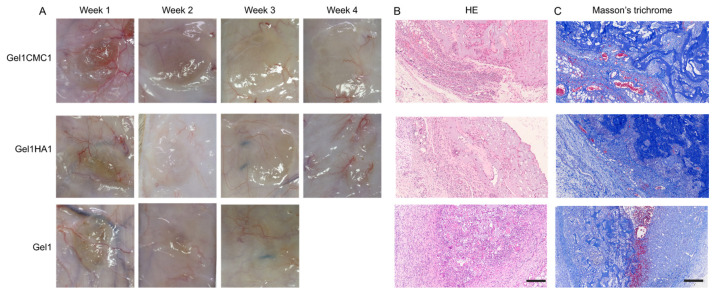
In vivo subcutaneous implantation of the hydrogels. (**A**). Gross images of planted hydrogels and surrounding tissue. (**B**). HE staining of samples at week 2. Scale bar: 200 μm. (**C**) Masson’s trichrome staining of samples at week 2. Scale bar: 200 μm.

To explore the potential applicability of the hydrogels in other medical scenarios that require angiogenesis, we used a subcutaneous implantation model in rats to evaluate tissue integration with hydrogel and neovascularisation [29]. This model could also reveal the foreign-body reaction and in vivo biocompatibility of the hydrogels. The gross images of subcutaneously implanted hydrogel and the surrounding tissue are shown in Figure 7A. The Gel1HA1 and Gel1CMC2 hydrogels completely disappeared within 4–5 weeks of implantation. The Gel1 hydrogel exhibited a degradation period of 2–3 weeks after implantation. Throughout the entire experiment, no significant adverse effects, rejection, infection, or obvious inflammation was detected. We further performed histological analysis on the 2-week samples. As shown in Figure 7B, the remaining hydrogel was found in sections, no obvious fibrotic capsule was detected, and fewer immune cells were found in the Gel1HA1 hydrogel group. These data indicate that immune response is minimal. Additionally, the new collagen deposition and vascular ingrowth, along with hydrogel degradation, were observed in conjunction with hydrogel degradation. These characteristics are essential for various soft tissue engineering and regeneration applications, making the hydrogel formulation a reliable alternative for addressing issues such as skin wound defects, atrophic scar, oral mucosal defects, and more. 

Mixtures of hydrogels composed of gelatine with various polysaccharides have been explored in previous studies, demonstrating potential utility in various tissue engineering strategies. However, it should be noted that for translational medicine, the formulation and screening processes should be consistent and of significant relevance. For example, researchers investigated widely used biomaterials like alginate, hyaluronic acid and gelatine in a variety of concentrations and hydrogel formulations to obtain a comprehensive understanding of their behaviours [20]. Nevertheless, the use of different crosslinking technologies resulted in distinct physiochemical properties. This limitation in turn impacts the conclusions drawn from comparative studies involving such materials. Thus, it is meaningful to assess the performance of different material composites under identical crosslinking reactions or the processing method. This approach helps to ensure a more meaningful and accurate comparison by eliminating the effects of crosslinking sites from different reaction methods. 

## 3. Conclusions

In conclusion, hydrogels formed by chemically crosslinking between gelatine and polysaccharides were successfully developed to function as tissue engineering scaffolds. These hydrogels exhibited favourable physiochemical properties, including suitable mechanical properties, appropriate swelling behaviour, and adequate pore structure, ensuring their compatibility with soft tissue engineering requirements. The in vitro cell experiments confirmed the biocompatibility and the ability of the promotion of cell proliferation. In the in vivo experiment, optimised hydrogels accelerated skin wound healing process by promoting re-epithelialisation, angiogenesis, and granulation formation, as evidenced by histological and immunochemical analysis. The subcutaneous implantation of the hydrogels in vivo demonstrated excellent tissue integration and neovascularisation, along with minimal immune response and favourable hydrogel biocompatibility. These findings present the hydrogel formulated in this study, demonstrating the advantageous features of each active component and highlighting the potential synergistic effects that can be harnessed for more effective clinical outcomes.

## 4. Materials and Methods

### 4.1. Materials

Gelatine (porcine, type B, ~225 g bloom) and carboxymethyl cellulose (50–200 mPa.s) were purchased from Sigma-Aldrich (Shanghai, China). Hyaluronic acid (Mw 10 kDa) was purchased from Focuschem, Qufu, China. N-(3-dimethylaminopropyl)-N-ethylcarbodiimide hydrochloride (EDC) was purchased from Sigma-Aldrich. All other chemicals were of analytical grade and used without further purification. Dulbecco’s Modified Eagle’s Medium (DMEM), foetal bovine serum (FBS), and penicillin/streptomycin were purchased from Invitrogen (Shanghai, China). 

### 4.2. Preparation of Different Scaffolds

All scaffold samples were prepared according to the concentrations described in Table 1.

To prepare the self-crosslinked Gel1 sample, gelatine was added to deionised water at 50 °C and stirred until fully dissolved. Then EDC/NHS solution was added to crosslink gelatine at room temperature for 24 h. The concentration of the EDC/NHS used in our study was 0.2% for EDC and 0.1% for NHS. 

To prepare Gel1CMC1 and Gel1CMC2 samples, gelatine was firstly dissolved in deionised water at 50 °C. The CMC solution was then added to the gelatine solution and stirred for full mixing. Subsequently, an EDC/NHS solution in MES was added to the mixture dropwise to crosslink the materials at room temperature for 24 h. The hydrogel precursor solutions were sterilized via filtration through a sterile filter with 0.22 μm, which could eliminate the microbial contaminants. The hydrogels were formed within a cylindrical plastic model with a diameter of 2.5 cm. 

### 4.3. Characterisation of the Hydrogel Scaffolds

Rheological properties were analysed with a TA rheometer equipped with an 8 mm parallel plate (*n* = 3). In total, 100 μL of the mixed hydrogel precursor was add on the plate and the test was performed at 25 °C. The hydrogel samples were tested using the following regimes: (1) time sweeps with a frequency of 1 Hz and a strain of 1%; and (2) strain sweeps ranging from 0.1 to 100% at 1 Hz. The storage modulus (G′) and loss modulus (G″) were monitored with time. G′ and G″ values on various strain sweeps were also measured. 

A swelling test was performed using freeze-dried hydrogel samples (*n* = 3), which were immersed in PBS at 37 °C for 48 h. The wet weight of the samples was measured, and the swelling degree was calculated as follows:

(Ws − W0)/W0 × 100%

where Ws is the hydrogel swollen mass and W0 is the freeze-dried mass.

### 4.4. Morphology Characterisation of the Hydrogel Scaffold

Scanning electron microscopy (SEM) was used to characterise the morphology and porosity of the freeze-dried hydrogels. The samples were cut in half using a razor blade and mounted onto an aluminium stub. Prior to imaging, the samples were coated with a layer of gold and examined using an AMETEK^®^ Quanta 3D FEG machine (Shanghai, China). ImageJ 1.53t software was used to determine the pore size of the hydrogel scaffold (*n* = 3).

### 4.5. In Vitro Cell Proliferation

3T3 cells were used to analyse the cytocompatibility and cell proliferation of the hydrogel scaffolds using CCK8 (Invitrogen). The hydrogel samples were set as experimental groups and were immersed in DI water to exclude the uncrosslinked agents.

All groups were added to each well and co-cultured with the cells in triplicate. Each hydrogel group was added to a 24-well plate (*n* = 3). Samples weighing 20 μg and a 10 μL cell suspension with a concentration of 1 × 10^6^ cells/mL were added to the top of the hydrogels and allowed to settle for 2 h before the addition of 1 mL full cell culture media to the well. Cells with a concentration of 10,000 cells per well were used as the control group and allowed to attach overnight. The cells were incubated at 37 °C and the test following standard protocol was performed at 24, 48, and 72 h after coculture. 

To visualise the cell status, a live/dead assay was used to confirm cell living status as the protocol described at 48 h, with calcein AM staining used for live cells (green colour) and ethidium homodimer-1 for dead cells (red colour). Images were taken using an inverted fluorescence microscope (Olympus IX81, Beijing, China).

### 4.6. Animal Experiment

Male C57BL/6 mice weighing 18–25 g were utilized to create an excisional skin wound model and to test the wound treatment efficacy. Sprague Dawley (SD) male rats 6–8 weeks in age with body weights ranging from 200 to 240 g were used for subcutaneous hydrogel implantation. The animals were fed ad libitum water and a rodent diet. All procedures were approved by the Experimental Animal Welfare Ethics Committee of Hangzhou Medical College (China). 

### 4.7. In Vivo Skin Wound Healing

Full-thickness excisional wounds with a diameter of 1 cm were created on the dorsal region of mice. Wounds were treated using Gel1CMC1, Gel1HA1, and saline (Blank), respectively. At regular time points, gross images of wounds were taken using a digital camera. The wound area was measured using ImageJ and the remaining percentage (%) was calculated as follows:

(Wt/W0) × 100%,

where Wt is the measured area at the regular time point and W0 is the original wound area (*n* = 5). 

Wound tissues were sampled at day 14 and fixed with 4% paraformaldehyde. Then, the samples were embedded in paraffin and cut into sections with 5 μm thickness. Hematoxylin and eosin (H&E, Sigma-Aldrich) were used to assess the tissue integrity and re-epithelialisation. Masson’s trichrome (Sigma-Aldrich) staining was conducted to detect the neo-dermis formation. In total, 10 sections were assessed per wound, and 5 wounds were examined per time point per group. 

Immunohistochemical staining of the sections was conducted. Sections were deparaffinized and washed three times in PBS for 5 min. Then, the sections were blocked with 5% serum for 30 min. Next, the slides were incubated in primary antibodies, VEGF (1:50), TNF-a (1:200), and IL-1β. 

Immunofluorescence was processed after the sections’ fixation in aceton at −20 °C. Sections were stained with antibodies of CD31 and α-SMA. After PBS washing, sections were incubated using secondary antibodies. The positive signals were further treated using DAB + kit (Dako, Glostrup, Denmark). For double immunofluorescent staining of CD31 and α-SMA, paraffin sections were first deparaffinized and washed with PBS. Then, the sections were blocked by serum and incubated with anti-CD31 primary antibody (Abbiotec, San Diego, CA, USA) and anti-α-SMA antibody (Abcam, Cambridge, UK). Lastly, sections were stained with DAPI (Abcam). Immunofluorescence images were taken via an AxioCam HRm camera mounted on a Zeiss Imager M2 microscope.

All tissues were imaged and five high-power microscopic fields for each separate wound were sampled by three blinded evaluators. The number of CD31^+^-stained vessels and the mature vessels stained by both CD31 and α-SMA were counted using ImageJ. 

### 4.8. In Vivo Tissue Integration and Vascularisation

The in vivo biocompatibility, tissue integration and effect of vascularisation of the hydrogels were evaluated in the subcutaneous implantation model in SD rats. The animals were allowed ad libitum feeding and were anesthetized via an intraperitoneal injection of 10% chloral hydrate. 

To create the model, four subcutaneous pockets were made on the dorsum of each animal by blunt dissection, with two on each side. One hydrogel weighing 200 mg was implanted in each pocket, and the incision was sutured with silk stitches. Implants were sampled every week to visualise adverse effects after implantation and degradability was observed until there was no obvious implant (five samples for each time point). At regular time points, animals were euthanised and implants were exposed by cutting the skin. Gross images of the implants were recorded to observe the remaining hydrogels. The samples of the hydrogel implants were fixed in a 4% formaldehyde/PBS solution for analysis. 

H&E and Masson’s trichrome were used to visualise the in vivo degradation, tissue integration, and neo-vessel formation of the samples following the manufacturer’s instruction for use. After staining, the prepared slides were examined under an optical microscope to gain insight into the structure and composition of the tissue. 

### 4.9. Statistical Analysis

The values are reported as mean ± standard deviation (SD). To determine statistical differences between the two groups, the unpaired Student’s *t* test was employed. A *p* value of less than 0.05 was considered statistically significant.

## Figures and Tables

**Table 1:** Composition of hydrogels.

Hydrogel Name	Gel (% *w*/*v*)	CMC (% *w*/*v*)	HA (% *w*/*v*)
Gel1	1.58	-	-
Gel1CMC1	1.58	0.1	-
Gel1CMC2	1.58	0.2	-
Gel1HA1	1.58	-	0.1
Gel1HA2	1.58	-	0.2

## Data Availability

Not applicable.
